# Polypyrrole Modified MoS_2_ Nanorod Composites as Durable Pseudocapacitive Anode Materials for Sodium-Ion Batteries

**DOI:** 10.3390/nano12122006

**Published:** 2022-06-10

**Authors:** Miao Jia, Tong Qi, Qiong Yuan, Peizhu Zhao, Mengqiu Jia

**Affiliations:** 1 College of Chemistry and Materials Engineering, Beijing Technology and Business University, Beijing 100048, China; 2 Beijing Key Laboratory of Electrochemical Process and Technology for Materials, Beijing University of Chemical Technology, Beijing 100029, China; qitongwork@163.com (T.Q.); 13716673969@163.com (Q.Y.); zhaopzhjy@163.com (P.Z.)

**Keywords:** metal sulfide, PPy, pseudocapacitive, sodium-ion battery, anode material

## Abstract

As a typical two-dimensional layered metal sulfide, MoS_2_ has a high theoretical capacity and large layer spacing, which is beneficial for ion transport. Herein, a facile polymerization method is employed to synthesize polypyrrole (PPy) nanotubes, followed by a hydrothermal method to obtain flower-rod-shaped MoS_2_/PPy (FR-MoS_2_/PPy) composites. The FR-MoS_2_/PPy achieves outstanding electrochemical performance as a sodium-ion battery anode. After 60 cycles under 100 mA g^−1^, the FR-MoS_2_/PPy can maintain a capacity of 431.9 mAh g^−1^. As for rate performance, when the current densities range from 0.1 to 2 A g^−1^, the capacities only reduce from 489.7 to 363.2 mAh g^−1^. The excellent performance comes from a high specific surface area provided by the unique structure and the synergistic effect between the components. Additionally, the introduction of conductive PPy improves the conductivity of the material and the internal hollow structure relieves the volume expansion. In addition, kinetic calculations show that the composite material has a high sodium-ion transmission rate, and the external pseudocapacitance behavior can also significantly improve its electrochemical performance. This method provides a new idea for the development of advanced high-capacity anode materials for sodium-ion batteries.

## 1. Introduction

With the continuous development of the commercialization of lithium-ion batteries, the scarce lithium resources on Earth can no longer meet the increasing demand for energy storage in the future; therefore, sodium-ion batteries have come into being [1,2,3]. Sodium belongs to the same group as lithium, thereby having many properties in common with lithium. However, the larger radius of Na^+^ compared to Li^+^ can easily lead to the accelerated crushing of electrode materials in the process of sodiation/desodiation [4,5]. Therefore, the development of suitable anode materials is particularly important.

Recently, two-dimensional layered metal chalcogenides have attracted extensive attention. Park et al. synthesized a variety of metal sulfides/selenides and achieved excellent performance in sodium-ion batteries [6,7,8]. Metal sulfides have good conductivity and large layer spacing, so they possess high theoretical capacities [9,10,11]. In addition, layered metal sulfides have unique structural advantages, namely a sandwich-like structure composed of sulfur and metal atoms (M-S-M); the layers are connected by covalent bonds, while each structure is connected by van der Waals force. This kind of structure can ensure rapid sodiation/desodiation [12,13]. Among them, MoS_2_ has a larger interlayer spacing (0.62 nm) compared to graphite, which is beneficial to accelerating kinetic processes. Li et al. [14] prepared flower-like MoS_2_, which showed remarkable performance in lithium-ion batteries. Kumar et al. [15] explored the binder effect on electrochemical capacity; the obtained MoS_2_ microflower presented 595 mAh g^−1^ under a current density of 50 mA g^−1^ with a Na-alginate binder. Yao et al. [16] synthesized grain-like MoS_2_ particulates by sulfuring MoO_3_; the unique structure could ease volume expansion. Although metal sulfide has its own advantages, there are still factors affecting battery capacity and cycle life, such as its low intrinsic conductivity, repeated electrochemical processes which lead to material structure crushing, formation of a solid electrolyte interface (SEI membrane) causing internal resistance, volume expansion, and so on. In order to solve these problems, researchers have obtained metal sulfide anode materials with good electrochemical properties by making materials at nanometer scale, compounding them with carbon, and designing the spatial structures of these materials [17,18,19,20,21]. Liu et al. [22] designed a type of MoS_2_ composite material wrapped in nitrogen-doped 3D hollow carbon framework pores, which can improve the stability of the structure and expose more active sites, such that it showed an excellent electrochemical performance of 413.3 mA h g^−1^ after 100 cycles at 0.1 A g^−1^. Wu et al. [23] synthesized Sn/MoS_2_@C double-walled hollow nanospheres; this hierarchical structure greatly increased the specific surface area and internal space of the composite materials, which could greatly alleviate the volume expansion of the materials during the charge and discharge processes, such that the composite exhibits excellent long-cycle stability in sodium-ion batteries and still has a capacity of approximately 432 mAh g^−1^ after 400 cycles at 1 A g^−1^. Therefore, the structural stability of MoS_2_ can be greatly improved through compounding, and the electrochemical performance can be enhanced.

Polypyrrole (PPy) has the advantages of high conductivity, a stable voltage window, environmental friendliness, low toxicity, good thermal stability, and excellent mechanical elasticity [24,25]. It could prevent oxidation of the electrode surface, build a conductive channel in the active electrode materials, and offer reversible redox reactions in the electrochemical processes, thereby attracting wild attention in electrochemical energy storage devices [26,27,28,29,30]. Composite materials of MoS_2_ and PPy have been most commonly used in supercapacitors. Tian et al. [31] used a simple method to guide PPy into an ordered molecular structure by in situ forming of MoS_2_ nanosheets on a 3D PPy frame, presenting huge application potential in supercapacitors. Hao et al. [32] prepared MoS_2_ nanosheets/PPy composites which were deposited on reduced graphene oxide (rGO) through a one-step hydrothermal method. Together, rGO and PPy could effectively improve the conductivity of MoS_2_; therefore, the composite material has excellent cycle stability and high energy density. In addition, Wang et al. [33] prepared CNT/MoS_2_@PPy composite materials; CNTs and PPy can provide effective ion transport channels, thereby reducing the electrical resistance during cycling and the volume expansion of the material, such that it exhibits excellent performance in lithium-ion batteries. Therefore, it can be expected that materials composed of MoS_2_ and PPy should also have broad application prospects in sodium-ion batteries.

Herein, a simple room-temperature polymerization method was used to synthesize PPy nanotubes, followed by a hydrothermal method to obtain the final hollow flower-rod-shaped MoS_2_/PPy nanotube composite materials (denoted as FR-MoS_2_/PPy). This material is expected to have an ultra-long cycle life and good rate performance.

## 2. Materials and Methods

### 2.1. Materials Synthesis

#### 2.1.1. Synthesis of PPy Nanotubes

A combination of 0.243 g of FeCl_3_ and 0.049 g of methyl orange was added to 30 mL of deionized water and, after stirring for 0.5 h, 100 μL of pyrrole was added. After stirring for 24 h at room temperature, PPy nanotubes were obtained, which were washed for several times with deionized water and dried in a vacuum oven at 50 °C.

#### 2.1.2. Synthesis of FR-MoS_2_/PPy

A combination of 0.1 g of as-prepared PPy nanotubes, 0.300 g of (NH_4_)_6_Mo_7_O_24_·4H_2_O, and 0.885 g of thiourea was stirred under ultrasound for 0.5 h and then added to 30 mL of deionized water. The resulting homogeneous solution was transferred into a 40 mL of Teflon-lined stainless-steel autoclave and heated at 180 °C for 24 h. The black precipitate was collected and washed several times with deionized water and ethanol, and the sample was dried at 50 °C for 10 h.

### 2.2. Materials Characterization

X-ray diffraction (XRD, D/max-2500B2+/PCX, Rigaku, Tokyo, Japan) was used to characterize the crystal structure of the as-prepared FR-MoS_2_/PPy, while X-ray photoelectron spectroscopy (XPS, ESCALAB 250, ThermoFisher Scientific, Waltham, MA, USA) was used for element valence analysis. A Fourier transform infrared spectrometer (FTIR, Nicolet iS50, Thermo Nicolet, Ramsey, MN, USA) was used to determine the functional groups of the conductive polymers. The content of MoS_2_ in the composite was tested by a thermogravimetric test (TGA, TGA/DSC 1/1100 SF, METTLER, Greifensee, Switzerland) at a temperature range of 25–800 °C. The morphology and element distribution of the composite material were tested by a scanning electron microscope (SEM, Supratm55, ZEISS, Oberkochen, Germany), its internal structure was observed by a transmission electron microscope (TEM, Tecnai G2 F30, FEI, Hillsboro, OR, USA), and the crystal structure of the material was further analyzed by a high-resolution transmission electron microscope (HRTEM, Tecnai G2 F30, FEI, Hillsboro, OR, USA) and a selected-area electron diffraction pattern (SEAD, Tecnai G2 F30, FEI, Hillsboro, OR, USA).

### 2.3. Electrochemical Measurements

To measure the electrochemical performance of the FR-MoS_2_/PPy composite material, a CR2025 coin battery was assembled with sodium as the counter electrode. The as-prepared composite material was mixed with a conductive agent (Super-P) and a binder (sodium alginate) with a ratio of 6:2:2, forming a uniform slurry. The slurry was coated uniformly on the copper foil, and then it was dried thoroughly in a vacuum at 120 °C for more than 10 h. The active-substance loading was about 1 mg cm^−2^. The electrolyte used was 1 M sodium perchlorate with 5% FEC dissolved in 1:1 ethylene carbonate and diethyl carbonate; an appropriate amount of FEC additive can not only significantly inhibit the decomposition of electrolyte solvents, but also improve the cycle stability of batteries. The cycle and rate performance were tested by a Neware CT3008 battery test system. Cyclic voltammetry (CV) was tested by a CS350 at different scan rates of 0.1–1 mV s^−1^, and electrochemical impedance spectroscopy (EIS) was performed at the same electrochemical workstation with a frequency ranging from 100 kHz to 0.01 Hz.

## 3. Results and Discussion

**Scheme 1:**
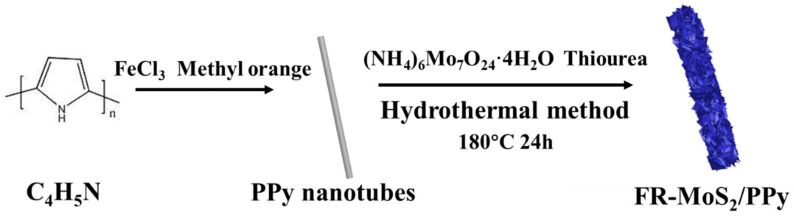
Schematic illustration of the synthesis process of FR-MoS_2_/PPy.

The preparation process of the FR-MoS_2_/PPy composite material is shown in Scheme 1. First, methyl orange and ferric chloride are used to initiate the polymerization of pyrrole monomers into nanotubes at room temperature. Then, the MoS_2_ nanosheets grow uniformly on the surface of the PPy nanotubes by a hydrothermal method. Finally, the MoS_2_ composite material is obtained.

**Figure 1:**
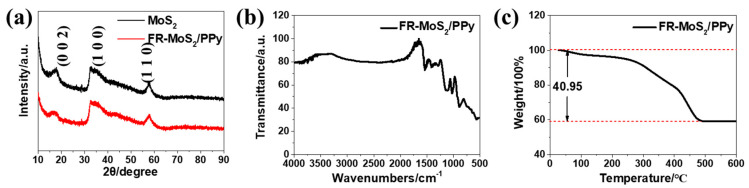
(**a**) XRD patterns of MoS_2_ and FR-MoS_2_/PPy, (**b**) FT-IR spectra, and (**c**) TGA curve of FR-MoS_2_/PPy.

It can be seen from the XRD pattern (Figure 1a) that the characteristic peaks of MoS_2_ before and after compounding with PPy have no significant changes. The peaks at about 16°, 33°, and 57° in the figure correspond to the (0 0 2), (1 0 0), and (1 1 0) crystal planes of MoS_2_, respectively, which proves that MoS_2_ forms a hexagonal phase structure (JCPDS 77–1716); the existence of the (0 0 2) plane can prove that MoS_2_ nanosheets grow uniformly on PPy [34].

The FT-IR result is presented in Figure 1b; the absorption peak at 1540 cm^−^^1^ represents the stretching vibration peak of C-C, the peak at 1400 cm^−1^ corresponds to the out-of-plane bending vibration peak of C-N, the peak at 1111 cm^−1^ corresponds to the in-bending vibration peak of =C-H, the peak at 1026 cm^−1^ corresponds to the in-plane bending vibration peak and out-of-plane bending vibration peak of N-H, the peak at 890 cm^−1^ corresponds to the out-of-plane bending vibration of =C-H, and the small peak at 1287 cm^−1^ is related to the doping state of PPy, all of which prove the successful combination of PPy and molybdenum sulfide [32,35,36]. Figure 1c shows the TGA result of FR-MoS_2_/PPy. After heating material from room temperature to 800 °C in an air atmosphere, 49.95% of the mass remains. Typically, the reaction of the composite material is as follows: 2MoS_2_ + 7O_2_ = 2MoO_3_ + 4SO_2_. Therefore, in addition to the transformation of PPy to CO_2_, the mass loss in the TGA curve also includes the SO_2_ loss in the reaction of MoS_2_, such that the equations can be listed as follows:

$$\frac{W(MoS_{2})}{M(MoS_{2})}=\frac{W(MoO_{3})}{M(MoO_{3})}$$

Among them, W(MoS_2_) and W(MoO_3_) represent the mass fraction of MoS_2_ and MoO_3_, and M(MoS_2_) and M(MoO_3_) represent the relative molecular masses of MoS_2_ and MoO_3_; therefore, the content of MoS_2_ in the composite material can be calculated as 55.6% [37].

**Figure 2:**
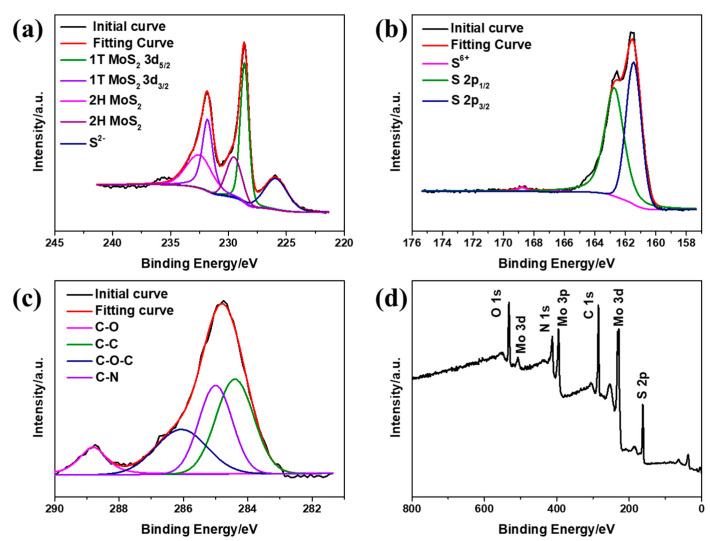
Peak-fitting curve of (**a**) Mo 3d, (**b**) S 2p, (**c**) C 1s, and (**d**) a survey of FR-MoS_2_/PPy.

The elemental composition and valence information are tested by XPS. Figure 2a shows the fitting peaks of the Mo element; two peaks at 228.9 and 232.1 eV can be seen, representing Mo 3d_5/2_ and Mo 3d_3/2_, respectively. They prove that there is Mo^4+^ in the composite material, which exists in the form of 1T-MoS_2_ in a metastable state with strong metallicity and good conductivity. The two peaks appearing at the higher binding energy area prove that the material contains part of the 2H-MoS_2_, which is a proper stable state. Figure 2b shows that the peaks at about 161.7 and 162.9 eV correspond to S 2p_3/2_ and S 2p_1/2_, respectively, which proves that S^2−^ exists in the composite material. The small peak near 168.7 eV corresponds to the part of the S^6+^ has not been fully reduced. Figure 2c shows the peak splitting of C 1s. It can be seen that there are four peaks at about 288.8, 286.4, 285, and 284.2 eV, corresponding to the existence of C-O, C-O-C, C-N, and C-C bonding, respectively. Figure 2d is the total spectrum of the composite material, which further proves the existence of nitrogen in the composite materials [38,39]. The above tests can prove the successful synthesis of the FR-MoS_2_/PPy.

**Figure 3:**
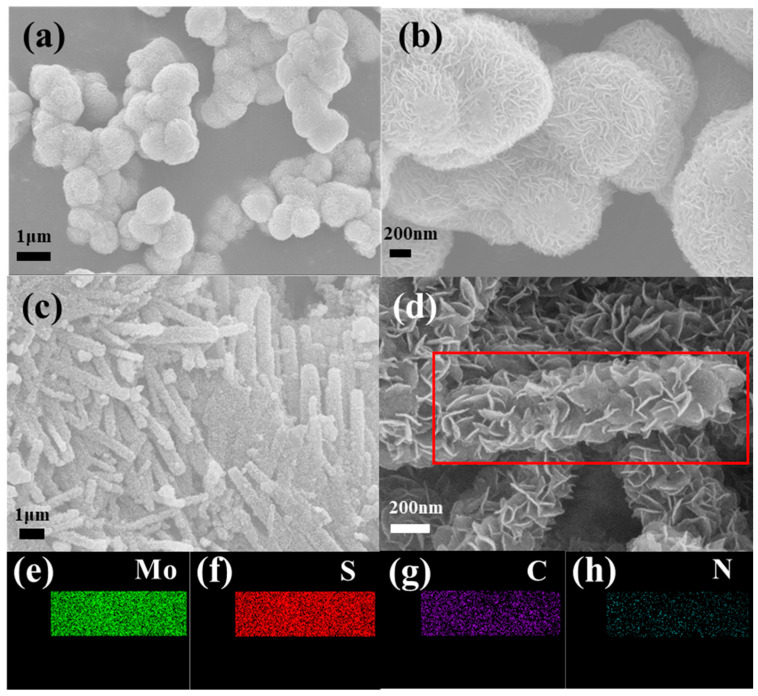
SEM images of (**a**,**b**) pure MoS_2_ and (**c**,**d**) FR-MoS_2_/PPy composite; EDS mapping analysis of (**e**) Mo, (**f**) S, (**g**) C, and (**h**) N.

Figure 3a,b show SEM images of pure MoS_2_ at different magnifications. It can be observed that the morphology of pure MoS_2_ presents a compact nanoflower structure which has a particle size of about 1 μm and is composed of numerous nanosheets. Figure 3c,d show that, after being compounded with PPy, a large number of MoS_2_ nanosheets are grown vertically on the PPy nanotubes, exhibiting a flower-rod morphology with a diameter of about 300 nm. It can be seen that the staggered arrangement of a large number of MoS_2_ nanosheets makes the material morphology more dispersed, so it can be expected to increase the specific surface area of the composite material, as well as increase the active sodium storage sites of the composite material and the sodium storage capacity. Figure 3e–h display the EDS mapping analysis of the FR-MoS_2_/PPy composites; the elements Mo, S, C, and N can be observed, further verifying the results of the XPS.

**Figure 4:**
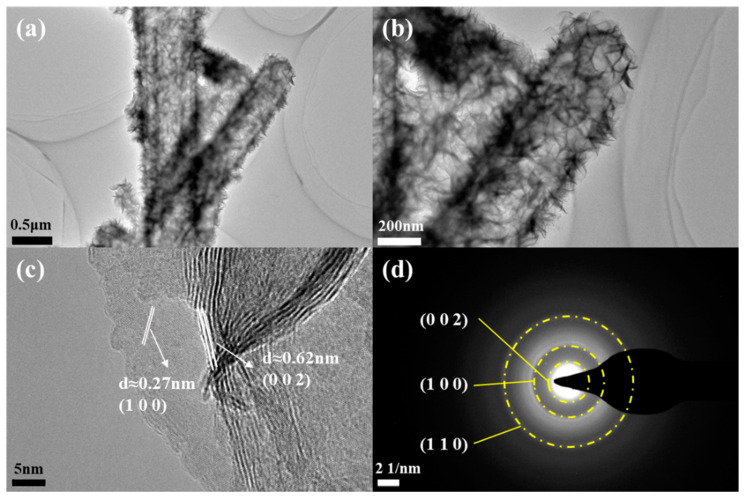
(**a**,**b**) TEM images, (**c**) HRTEM, and (**d**) SAED pattern of FR-MoS_2_/PPy.

It can be seen from the TEM results (Figure 4a,b) that the composite material, with a diameter of about 300 nm, has a hollow structure, with PPy nanotubes inside and MoS_2_ nanosheets uniformly coated on the outside. The HRTEM results (Figure 4c) show two different crystal plane spacings of 0.62 and 0.27 nm, corresponding to the two crystal planes (0 0 2) and (1 0 0) of MoS_2_, respectively. The SAED pattern (Figure 4d) can be observed showing three clear diffraction rings, corresponding to the three crystal planes (0 0 2), (1 0 0), and (1 1 0) of MoS_2_ [40,41].

**Figure 5:**
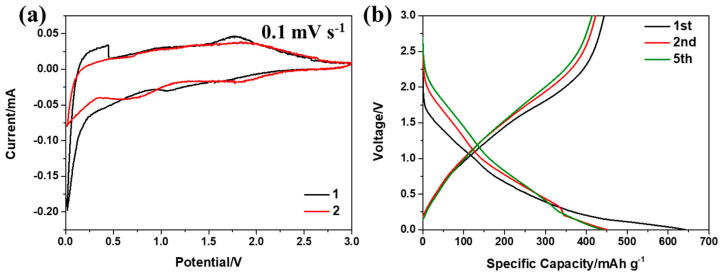
(**a**) CV curves under a scan rate of 0.1 mV s^−1^ for the first two cycles; (**b**) charge-discharge curve of 1st, 2nd, 5th cycles at a current density of 100 mA g^−1^.

As shown in Figure 5a, the CV curves of the FR-MoS_2_/PPy were measured at a scan rate of 0.1 mV s^−1^. It can be seen that, during the first cathodic scan, the small reduction peak at 1.1 V corresponds to the beginning stage of the sodium ion insertion among the MoS_2_ layers and the formation of the SEI film. The reduction peak at 0.1 V corresponds to the conversion reaction of MoS_2_ to Mo [38]. In the anode process, the oxidation peak at about 1.7 V corresponds to the conversion of elemental Mo to MoS_2_, which proves the reversibility of the electrode reaction during the electrochemical cycle [16]. In the second cycle, the reaction peak of the electrode shifts slightly, which is due to the polarization phenomenon that cannot be avoided during the battery reaction [42]. The reaction formula can be written as follows [43]:

MoS_2_ + 4Na^+^ + 4e^−^ ↔ Mo + 2Na_2_S

Both pure MoS_2_ and FR-MoS_2_/PPy share the same sodium storage mechanism. As shown in Figure 5b, at a current density of 100 mA g^−1^, the initial discharge capacity of the FR-MoS_2_/PPy composite is 642.7 mAh g^−1^, and the initial charge capacity is 443.5 mAh g^−1^. After the second cycle, the discharge curves gradually overlap, indicating that the composite material has excellent cycle stability.

**Figure 6:**
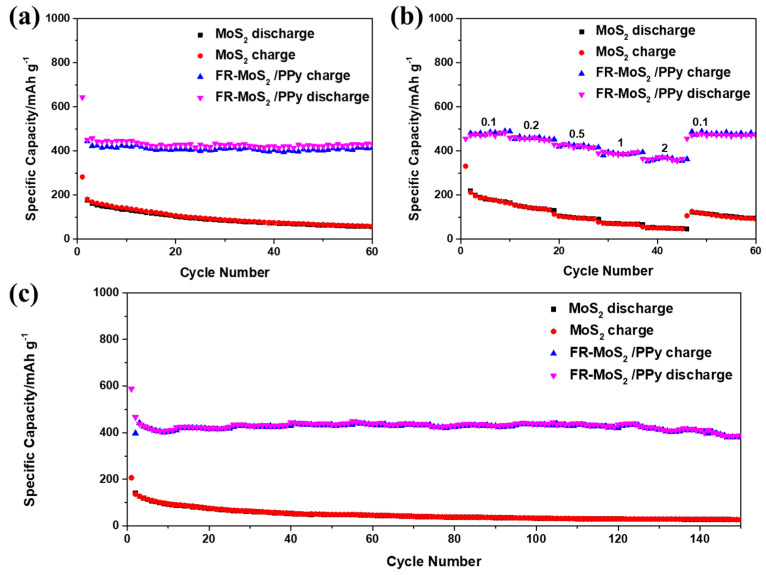
(**a**) Cycle performance under 100 mA g^−1^, (**b**) rate performance, and (**c**) cycle performance under 500 mA g^−1^ of MoS_2_ and FR-MoS_2_/PPy.

Figure 6 shows the electrochemical performance of pure MoS_2_ and FR-MoS_2_/PPy composite materials. As shown in Figure 6a, after 60 cycles at a current density of 100 mA g^−1^, the FR-MoS_2_/PPy can still maintain a stable capacity of 431.9 mAh g^−1^, showing excellent cycle stability, while the capacity of the pure material shows a declining trend, and only a capacity of 58.5 mAh g^−1^ remains after 60 cycles. Figure 6b shows the rate performance; when FR-MoS_2_/PPy cycles 10 times under current densities of 0.1, 0.2, 0.5, 1, and 2 A g^−1^, the capacities are 489.7, 452.8, 416.2, 394.5, and 363.2 mAh g^−1^, respectively, showing excellent rate performance, and when the current density returns to 0.1 A g^−1^, the capacity can be stably maintained at 475.2 mAh g^−1^. However, under the same current density as with FR-MoS_2_/PPy, the rate performance of the pure material is relatively inferior, maintaining capacities of only 168.7, 133.5, 90.7, 66.0, and 47.4 mAh g^−1^, and when the current density returns to 0.1 mA g^−1^, the capacity can only reach 94.7 mAh g^−1^. The cycle stability of the two materials was also tested under a relatively high current (500 mA g^−1^); as shown in Figure 6c, FR-MoS_2_/PPy and pure MoS_2_ can maintain 386.9 and 27.4 mAh g^−1^ after 150 cycles, respectively. The above results show that the compounding with PPy helps MoS_2_ gain higher capacity and more stable performance than pure MoS_2_, which could benefit from the synergistic effect between the two compounds and the enhanced electrochemical performance brought by PPy.

**Figure 7:**
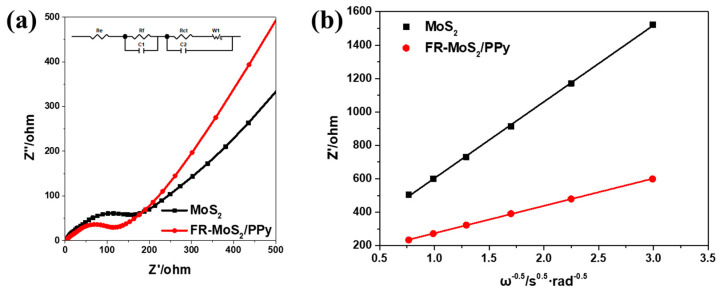
(**a**) EIS plots of MoS_2_ and FR-MoS_2_/PPy after 20 cycles under 200 mA g^−1^, (**b**) Z′ vs. ω^−0.5^ plots calculated from EIS under low frequency.

The EIS test is performed after 20 cycles under a current density of 200 mA g^−1^; the fitting results can be used to judge the improvement of the electroconductivity and charge transfer rate of the composite. As shown in Figure 7a, the impedance curves of MoS_2_ and FR-MoS_2_/PPy are both made up of semicircles and inclined straight lines, indicating that the electrochemical kinetics of the materials can be divided into two steps. First, sodium ions migrate through the electrolyte to reach the vicinity of the electrode surface, such that the external circuit transfers electrons to the electrode surface to maintain charge balance. In this period, the semicircle of the EIS represents the formation of SEI layer impedance (R_f_), charge transfer impedance (R_ct_), and double capacitance impedance (C). Then, a large amount of sodium ions can accumulate on the electrode surface. Therefore, the Na^+^ concentration on the electrode surface becomes much higher than that of the interior. The resulting Na^+^ concentration gradient can cause sodium ions to diffuse from the electrode surface to the interior to maintain balance. This process produces a diffusion impedance, called “Warburg impedance” (W_s_), which can be reflected by the slash part of the EIS results. In addition, R_e_ represents the impedance of the battery, which can be reflected in the intercept of the high-frequency region Z’ [44]. The fitting results of R_e_, R_f_, and R_ct_ for the MoS_2_ and FR-MoS_2_/PPy electrodes are shown in Table 1. It can be clearly seen that the R_ct_ values of MoS_2_ and FR-MoS_2_/PPy are 105.60 and 75.06 Ω, respectively. The charge transfer resistance of FR-MoS_2_/PPy is significantly reduced compared to that of pure MoS_2_, owing to the introduction of PPy increasing the electronic conductivity of composite material, which is beneficial to improving the electrochemical performance of material. Table 2 shows the electrochemical performance comparison of different MoS_2_ composite materials.

In addition, the fitting results in the low-frequency region can be used to calculate the diffusion coefficient (D_Na_) of Na^+^, and the calculation equations can be written as follows [45,46]:

$$Z^{\prime }=R_{e}+R_{\mathrm{ct}}+{\sigma \omega }^{-0.5}$$

$$D_{{\mathrm{Na}}^{+}}=\frac{{\left(\mathrm{RT}\right)}^{2}}{2{\left({\mathrm{An}}^{2}F^{2}C\sigma \right)}^{2}}$$

Among them, R represents the ideal gas constant with a value of 8.314, T represents the absolute temperature in Kelvin, with a value of 298.15 K, and n represents the number of electrons transferred in the electrochemical process. F is the Faraday constant with a value of 96,500 C mol^−1^. C is the sodium ion concentration. Ω stands for the angular frequency and σ represents the Warburg factor, which can be calculated from Z′ fitting. Figure 7b shows the Z′-ω^−0.5^ graph. The sodium diffusion coefficients of the MoS_2_ and FR-MoS_2_/PPy are 7.23 × 10^−11^ and 1.07 × 10^−10^ cm^2^ s^−1^, respectively, indicating that FR-MoS_2_/PPy has a higher ion transfer rate.

**Figure 8:**
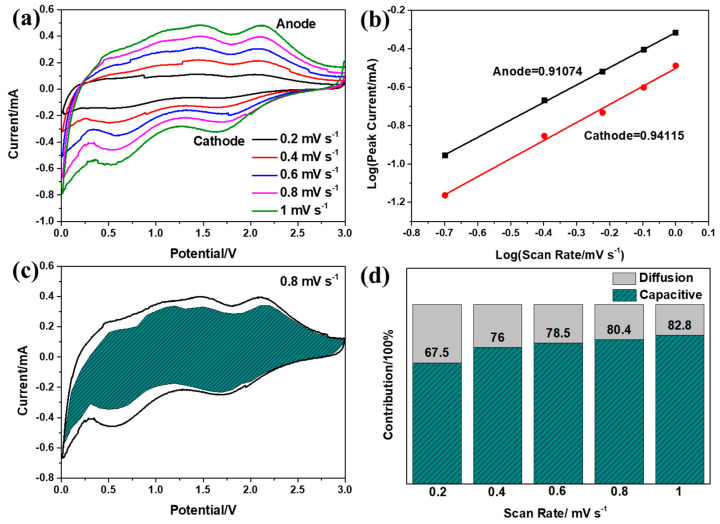
(**a**) The second-cycle CV curves of the FR-MoS_2_/PPy electrode under different scan rates, ranging from 0.2 to 1 mV s^−1^; (**b**) the slope of the fitted curve of Log (scan rate) and Log (peak current); (**c**) the proportion of the pseudocapacitance contribution during the electrochemical process when the scan rate is 0.8 mV s^−1^; (**d**) proportion of the pseudocapacitance contribution at different scan rates.

In order to further study the characteristics of the sodium storage processes of the FR-MoS_2_/PPy electrode, the CV curves were tested under scan rates ranging from 0.2 to 1 mV s^−1^ (Figure 8a). The following equation can express the relationship between Log (scan rate, mV s^−1^) and Log (peak current, A):

i = av^b^

Log(i) = bLog(v) + Log(a)

In the above formula, when the b value is close to 0.5, ion diffusion determines the electrochemical reaction, and when the b value is close to 1, it is mainly controlled by the pseudocapacitance process [46,47]. The linear relationship between Log (i) and Log (v) is shown in Figure 8b. The corresponding b values of the two main redox peaks are 0.91074 for the anode and 0.94115 for the cathode, respectively, which indicates that the redox process of the FR-MoS_2_/PPy composite includes partial pseudocapacitive performance, leading to its relatively remarkable rate performance. The total current at a fixed potential includes the pseudocapacitance mechanism (k_1_v) and the ion diffusion process (k_2_v^1/2^), which can be calculated by the following formula:

i = k_1_v + k_2_v^1/2^

Here, k_1_v and k_2_v^1/2^ represent the pseudocapacitance process and the diffusion-controlled Na^+^ insertion–extraction, respectively [48]. Figure 8c shows the ratio of the pseudocapacitance process at a scanning speed of 0.8 mV s^−1^. It can be seen that the ratio of the pseudocapacitance contribution is about 80.4% (green shaded area). Figure 8d shows that the contribution rates of the pseudocapacitance were 67.5%, 76%, 78.5%, 80.4%, and 82.8% at different scanning speeds of 0.2, 0.4, 0.6, 0.8, and 1 mV s^−1^. This means that, as the scanning speed increases, the contribution of the pseudocapacitance gradually accounts for more of the total capacitance.

## 4. Conclusions

Herein, a hollow MoS_2_/PPy composite material with flower-rod morphology (FR-MoS_2_/PPy) was successfully synthesized through a two-step method. The as-prepared material obtained outstanding performance in electrochemical tests. Under a current density of 100 mA g^−1^, the FR-MoS_2_/PPy can maintain a capacity of 431.9 mAh g^−1^ after 60 cycles, and even under a high density of 2 A g^−1^, the composite can still reach a high capacity of 363.2 mAh g^−1^. The excellent electrochemical performance can be attributed to the synergistic effect between the different components of the composite material. The hollow structure also relieves the volume expansion of the material during the deintercalation of the sodium. In addition, the dispersed MoS_2_ nanosheets can increase the specific surface area of the material, to a certain extent, and increase the active sites of sodium storage. Furthermore, the pseudocapacitance characteristics can effectively increase the energy density of the electrode material so as to obtain excellent rate performance.

## Figures and Tables

**Table 1:** EIS fitting results of MoS_2_ and FR-MoS_2_/PPy.

Samples	R_e_/Ω	R_f_/Ω
MoS_2_	5.13	25.14
FR-MoS_2_/PPy	5.54	18.46

**Table 2:** Comparison of different MoS_2_ anode materials in sodium-ion batteries.

Samples	Current Density (mA g^−1^)	Cycle Number	Capacity (mAh g^−1^)	Reference
MoS_2_ microflowers	100	50	595	[15]
G-MoS_2_	500	300	312	[16]
MoS_2_ hollow spheres	100	100	391.4	[17]
FR-MoS_2_/PPy	100	60	431.9	This Work
	500	150	386.9	

## Data Availability

The data presented in this study are available upon request from the corresponding author.
